# Re-evaluating the gender gap: a cross-sectional analysis of accepted American Academy of Neurology annual meeting abstracts in 2020 and 2021

**DOI:** 10.3389/frma.2024.1360367

**Published:** 2024-04-11

**Authors:** Minseon Kim, Youngran Kim, Anjail Z. Sharrief, Thy P. Nguyen

**Affiliations:** ^1^Department of Neurology, University of Texas Houston Medical School, Houston, TX, United States; ^2^Center for Healthcare Data, University of Texas Houston School of Public Health, Houston, TX, United States

**Keywords:** gender, disparity, neurology, abstracts, gender gap

## Abstract

**Background and objective:**

Prior studies reveal that invited speaker panels, editorial boards, authors of practice guidelines, and senior authors of published articles are disproportionately male in the neurology field. We aimed to analyze a gender gap in authorship of accepted abstracts to the American Academy of Neurology annual meetings in 2020 and 2021.

**Design/methods:**

This is a cross-sectional study evaluating the proportions of female first and senior abstract authors in 2020 and 2021. Abstracts were reviewed manually (*n* = 3,211 in 2020; *n* = 2,178 in 2021). Data were collected regarding the gender of first and senior authors, subspecialties, and origin of research (USA, international, or corporate-affiliated). Then, we compared the percentages of female first and senior authors in the 2 years to assess for any short-term effects of the COVID-19 pandemic.

**Results:**

Accepted abstracts with female first and senior authors comprised 46%, 34% in 2020, and the same in 2021, without change. Female senior authors had a significantly higher proportion of female first authors than their male senior author counterparts. The analysis of subspecialties with more than 100 abstracts showed the lowest percentages of female senior authors was oncology (24.7%), sleep (25.5%), headache (28.7%), and cerebrovascular disease (29%) in 2020. Cerebrovascular disease (29%) and behavioral neurology (24.7%) had the lowest percentage of female senior authors in 2021. In the analysis of the origin of research, corporate-affiliated authors had the lowest percentages of female first (34 and 36%) and senior authors (22.6 and 27.6%).

**Conclusion:**

The gender gap in neurology was reaffirmed in regards to female senior authorship overall and in subgroups of abstracts including cerebrovascular disease, headache, behavioral neurology, sleep, oncology, and corporate-affiliated research.

## Introduction

The gender gap in neurology and other academic fields has been established. In 2021, women composed 55% of matriculating medical students and 47.9% of neurology residents. Overall, practicing neurologists in 2021 were 31% women. Neurology medical school faculty were 43% women (AAMC, 2022a,b). However, the proportion of female faculty dwindled with advancing rank in academia, suggesting attrition or lack of promotion. Female instructors comprised 59%, assistant professors 50%, associate professors 42%, and professors only 25%. There were only 23 female chairs in 134 neurology departments, comprising 17% women. These percentages for rank were similar to those for other specialties. However, neurology lagged for the percentage of female chairs, which is 23% in all academic departments (AAMC, 2022b).

Considerations for academic promotion include productivity, leadership roles, publications, grants, and professional regard. Previous studies have shown a gender gap in neurology, including invited speaker engagements, senior authors of publications, authors of practice guidelines, editorial boards, and compensation (Jensen, 2018; Pakpoor et al., 2018; Fournier et al., 2020; Mariotto et al., 2020; Nguyen et al., 2021; Yu et al., 2022; Ross et al., 2023). An analysis of 29 top-ranked neurology programs' faculty revealed that men had more publications than women at every rank (McDermott et al., 2018; AAMC, 2019). Women represented 29% of invited speakers to an international stroke conference (Fournier et al., 2020). In a 35-year analysis of three high-impact neurology journals, 18% of senior authors were women (Pakpoor et al., 2018). In an analysis of 68 practice guidelines with 709 authors, 18% had women as first authors (Ross et al., 2023). Only 11% of the editorial boards of neurology journals have women as editors-in-chief (Mariotto et al., 2020). Women neurologists were paid 10% less than their male counterparts, even after accounting for call status and subspecialties (Yu et al., 2022). Four key gatekeepers were identified as playing a key role in gender disparity/equity in neurology; medical schools/academic centers, funding sources, medical journals, and medical societies (Silver, 2019).

The American Academy of Neurology (AAN) is the largest international society of neurologists and neuroscience professionals, comprising 38,000 members in 2020. Prior annual AAN meeting attendance ranged from 10,000 to 15,000. The practice type of members and subspecialties is diverse. The analysis of accepted abstracts at this national conference was evaluated due to the diverse representation and large number of accepted abstracts. We aimed to evaluate a gender gap in accepted AAN abstracts.

The coronavirus pandemic also had effects on the gender gap. A survey across a wide range of science fields in the United States and Europe evaluated the impact of the pandemic on working hours and time allocations. The results showed time devoted to research declined by 24% during the pandemic. Female scientists reported a 5% larger decline in research time and having a child age 5 years old or younger was associated with a 17% larger decline (Myers et al., 2020). In the private sector, a survey revealed that 25% of women considered quitting their jobs, reducing their hours, or changing careers during the pandemic (Coury et al., 2020; Higginbotham, 2020). Another survey of academic medicine faculty with 1,186 respondents at a single institution revealed that women were more likely than men to consider leaving or reducing employment (28 vs. 12%). Women with children were more likely to consider leaving than women without children (35 vs. 17%). Women were also more likely to decline leadership opportunities than men (29 vs. 13%; Matulevicius et al., 2021). Although variable depending on discipline, female scientists published fewer papers and had fewer citations during the pandemic than the year prior (Amano-Patiño et al., 2020; Andersen et al., 2020; Wehner et al., 2020). Female first and corresponding authors also decreased (Andersen et al., 2020; Fry et al., 2020; Wehner et al., 2020; Matulevicius et al., 2021). We hypothesized that the proportion of female first and senior authors in accepted AAN abstracts would decline during the pandemic.

## Methods

### Standard protocol approvals, registrations, and patient consents

This research was submitted to the University of Texas Houston IRB and deemed non-regulatory, as it did not meet the definition for human subjects research.

This was a cross-sectional study evaluating the proportions of female first and senior authors of accepted abstracts during 2 years of the COVID-19 pandemic.

The accepted 2020 and 2021 AAN scientific abstracts were previously available online (see Data Availability). The 2020 AAN abstract deadline was 21 October 2019, prior to the COVID-19 pandemic. The outbreak of COVID-19 was first reported from Wuhan, China on 31 December 2019, and worldwide by the spring of 2020. The 2021 AAN abstract deadline was 10 October 2020, during the pandemic.

The inclusion criterion was all accepted abstracts in 2020 and 2021. There were 3,211 abstracts from 2020 and 2,178 abstracts from 2021. These abstract years were chosen to represent the years during the pandemic. We excluded abstracts in which gender could not be verified through public academic profiles, social media profiles, and/or the use of pronouns. For the purposes of this study, we considered gender as male or female. Non-binary gender could not be consistently assessed due to our methods of manual verification.

**Figure 1:**
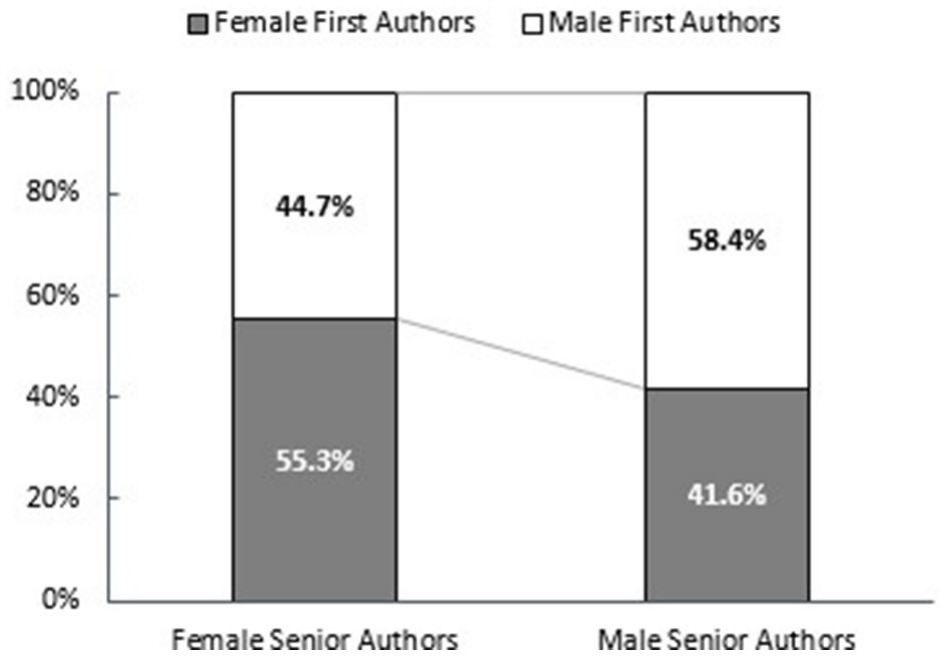
Proportion of female first authors depending on gender of senior author.

We collected data on the primary variable of interest, the gender of the first and last authors. The last author was presumed to be the senior author, based on convention. We also collected any author's affiliation with a corporation, subspecialty category, and origin of abstract (USA, international, or corporate). A corporation was classified as pharmaceutical, technology/device, data analytics company, or non-profit organization. For a limited number of names (*n* = 10), verification of gender could not be performed by the means above, and they were excluded from the analysis. Subspecialty categories with more than 100 abstracts were included. All abstracts were independently reviewed by two authors. For a limited number of names (*n* = 10), verification of gender could not be performed by the means above, and they were excluded from the analysis. Differences in the proportions of female authors were compared between 2020 and 2021, and statistical significances were determined using chi-square tests. Female first and senior authorship were examined across subspecialties using pooled data of both years, compared to the overall average. We also analyzed the proportion of female first authors depending on the gender of senior authors (Figure 1).

### Data availability

The accepted 2020 and 2021 AAN scientific abstracts were previously available online. Information about the meetings are available at the following links.

https://www.neurology.org/toc/wnl/96/15_supplement
https://www.aan.com/events/2020-annual-meeting.

## Results

There were 3,211 abstracts from 2020 and 2,178 abstracts from 2021. The percentages of female first and senior authors for accepted overall abstracts did not significantly change from 2020 to 2021. Female first authors were 46.1 and 46.7%, respectively. Female senior authors were 34.2 and 34.8% (2020 and 2021; Table 1). The proportion of female first authors was higher if the senior author was also female (55.3 vs. 41.6%, *p* < 0.001; shown in Figure 1).

**Table 1:** Proportion of female first and senior authors for accepted abstracts to AAN in 2020 and 2021.

	**No. of female first authors/subtotal (%)**			**No. of female senior authors/subtotal (%)**		
	**2020**	**2021**	***p*** **-value**	**2020**	**2021**	***p*** **-value**
**Overall**	1,480/3,211 (46.1)	1,018/2,178 (46.7)	0.64	1,099/3,211 (34.2)	759/2,178 (34.8)	0.64
**Origin of study**						
Domestic	959/1,841 (52.1)	673/1,314 (51.2)	0.63	687/1,841 (37.3)	493/1,314 (37.5)	0.91
International	338/839 (40.3)	196/429 (45.7)	0.065	292/839 (34.8)	146/429 (34.0)	0.79
Corporate	183/531 (34.5)	149/435 (34.3)	0.95	120/531 (22.6)	120/435 (27.6)	0.07
**Subspecialty**						
Multiple Sclerosis	193/439 (44.0)	123/271 (45.4)	0.71	163/439 (37.1)	110/271 (40.6)	0.36
Cerebrovascular	173/417 (41.5)	139/283 (49.1)	0.046	122/417 (29.3)	82/283 (29.0)	0.94
Movement Disorders	140/323 (43.3)	95/201 (47.3)	0.38	113/323 (35.0)	71/201 (35.3)	0.94
Neuromuscular	117/268 (43.7)	69/170 (40.6)	0.53	93/268 (34.7)	54/170 (31.8)	0.53
Epilepsy	109/223 (48.9)	74/162 (45.7)	0.54	86/223 (38.6)	54/162 (33.3)	0.29
Headache	81/202 (40.1)	67/159 (42.1)	0.70	58/202 (28.7)	51/159 (32.1)	0.49
Autoimmune	95/211 (45.0)	56/127 (44.1)	0.87	69/211 (32.7)	45/127 (35.4)	0.61
General Neurology	68/148 (45.9)	41/104 (39.4)	0.30	49/148 (33.1)	36/104 (34.6)	0.80
Dementia	70/143 (49.0)	52/102 (51.0)	0.75	51/143 (35.7)	33/102 (32.4)	0.59
Pedi Neuro	77/132 (58.3)	50/84 (59.5)	0.86	59/132 (44.7)	40/84 (47.6)	0.67
Infectious Disease	55/103 (53.4)	54/104 (51.9)	0.83	40/103 (38.8)	34/104 (32.7)	0.36
Neurocritical Care	39/75 (52.0)	31/63 (49.2)	0.74	30/75 (40.0)	22/63 (34.9)	0.54
Oncology	34/73 (46.6)	19/52 (36.5)	0.26	18/73 (24.7)	16/52 (30.8)	0.45
Education/Research/Methods	47/72 (65.3)	37/49 (75.5)	0.23	29/72 (40.3)	26/49 (53.1)	0.17
Beh. Neuro	41/69 (59.4)	23/50 (46.0)	0.15	21/69 (30.4)	12/50 (24.0)	0.44
Sleep	14/47 (29.8)	12/33 (36.4)	0.54	12/47 (25.5)	10/33 (30.3)	0.64
Neuro-Ophtho/Otology	21/49 (42.9)	12/29 (41.4)	0.90	16/49 (32.7)	14/29 (48.3)	0.17
Policy	28/47 (59.6)	14/26 (53.8)	0.64	21/47 (44.7)	08/26 (30.8)	0.25

**Figure 2:**
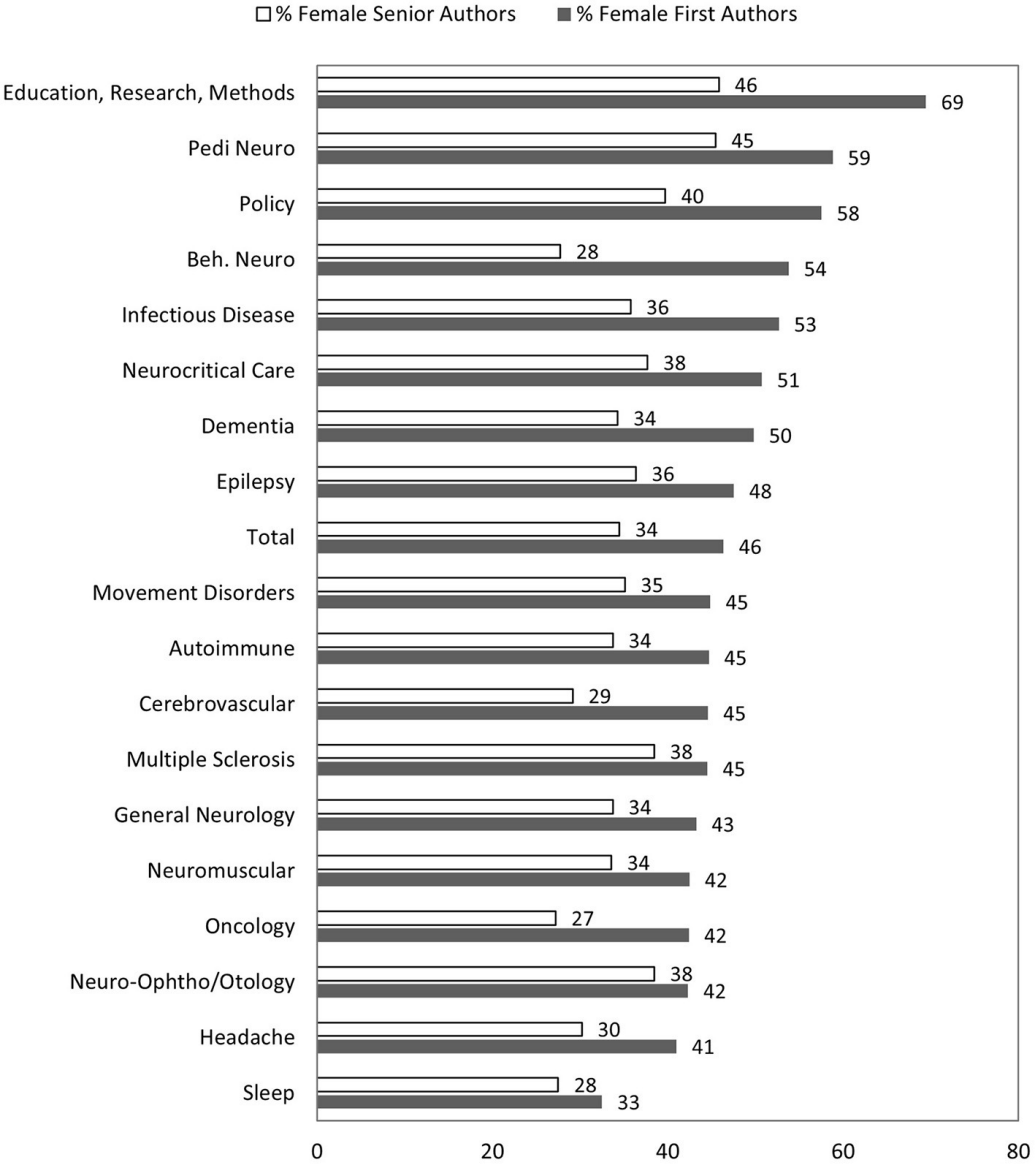
Proportions of female first and senior author by subspecialty (pooled data of 2020 and 2021 abstracts).

We also analyzed the percentages of female first and senior authors based on subspecialties. In the analysis of subspecialties with more than 100 abstracts, the percentage of female first authors remained similar across 2 years except for cerebrovascular disease, which increased from 41.5 to 49.1%, *p* = 0.046 (Table 1). The lowest percentages of female senior authors were oncology (24.7%), sleep (25.5%), cerebrovascular disease (29.2%), and headache (28.7%) in 2020 (Table 1). In pooled data of 2020 and 2021 abstracts, subspecialties of education research methods, pediatric neurology, and policy had significantly more female first and senior authors than overall average (Figure 2).

In the analysis of the origin of research, abstracts were classified based on whether the first author's affiliation was domestic (USA), international, or corporate (if any contributing author's primary affiliation was a corporation). The lowest percentages of female first (34.5 and 34.3%) and senior authors (22.6 and 27.6%) were noted in corporate-affiliated abstracts.

## Discussion

Our research on the accepted abstracts identified a gender gap, particularly with female senior authors. The under-representation of 31% female senior abstract authors (compared to 43% neurology academic women faculty) could have downstream implications. These include less networking, leadership opportunities, publications, citations (due to the lack of dissemination of research), and less influence on younger neurologists (Cumberworth et al., 2012).

We did not identify a change in the proportion of female authors during the pandemic. A confounder of our analysis was the blinding of abstracts to reviewers during the timeframe studied. Prior studies have shown that blinding reduces bias in abstract selection, but not specifically gender bias (Ross et al., 2006). An additional confounding factor is the introduction of a virtual option to the analyzed meetings. Virtual meetings may be more accessible to specific attendees. Virtual meetings may appeal to those on parental leave, with more clinical demands, financial constraints, international physicians, and parents of young children (Vervoort et al., 2021). Another consideration is the temporary, nationwide shutdown of elective procedures and in-person, outpatient, clinical care may have led to less clinical time or more flexibility in clinical time, leading to more time to devote to abstract preparation and submission.

In subspecialty analysis, education research methods had a significantly higher proportion of female first and senior authors compared to the overall average. This may be due to increasing proportions of early career women in academic medicine (59% of instructors and 48% of assistant professors) and clinician–educator tracks (AAMC, 2022b).

Our study identified a positive impact of female mentors (senior authors). A retracted Nature article proposed that female mentors and mentees do not benefit from a same-gender mentor–mentee relationship in terms of future productivity. Our cross-sectional analysis revealed that female senior authors had a higher proportion of female first authors than their male counterparts in 2020 and 2021 (Figure 1). A similar relationship was identified, as male senior authors had a higher proportion of male first authors (Figure 1). This finding was also noted in the business realm, with 71% of sponsors identifying as the same gender or race as their sponsor (CTI, 2019). Additionally, a single female leader or moderator for conference sessions was associated with a higher percentage of female speakers (Galloway et al., 2020).

Our research was limited as we included a single national conference and analyzed the accepted abstracts over 2 years. Tracking changes in authorship over several years would identify trends that are more meaningful than a comparison of the 2 years. We also did not analyze submitted abstracts. Other limitations are manual verification of gender rather than self-reporting. Gender was viewed as binary. Given that the abstracts do not identify the corresponding author, the last author may not be the senior author. Co-first male and female authors could not be determined. Furthermore, we did not collect data on trainee status and did not have information on the number and age of dependents. Another limitation is a lack of information on race, ethnicity, and socioeconomic status.

## Conclusion

Although the COVID-19 pandemic has disproportionately impacted female scientists in many fields, our study did not show a change in overall proportion of female first and senior abstract authorships in the 2 years analyzed. However, a larger study analyzing abstracts in multiple years before, during, and after the pandemic would more accurately assess the impact of COVID-19 on gender changes of authorship. A gender gap was reaffirmed in the overall proportion of female senior authors compared to women neurologists in academic medicine. Corporate-affiliated research also had lower percentages of female first and senior authors. Prior studies have suggested that financial support, flexible work schedule/promotion timeline, and advocacy on gender equity at the institutional and national levels are crucial to reducing the gender gap in academic and clinical research (Galloway et al., 2020; Davis et al., 2022).

## Data availability statement

The original contributions presented in the study are included in the article/supplementary material, further inquiries can be directed to the corresponding author.

## Author contributions

MK: Data curation, Formal analysis, Writing – original draft. YK: Formal analysis, Methodology, Writing – review & editing. AS: Writing – review & editing. TN: Conceptualization, Data curation, Formal analysis, Methodology, Supervision, Writing – original draft, Writing – review & editing.
